# Understanding young adults’ perceptions regarding human papillomavirus vaccination: A qualitative study

**DOI:** 10.1371/journal.pone.0323063

**Published:** 2025-05-12

**Authors:** Oluwafemifola Oyedeji, Carman North, Kristina W. Kintziger, Samantha Ehrlich, Jill Maples, Justin Gatwood, Cristina S. Barroso

**Affiliations:** 1 Department of Public Health, The University of Tennessee, Knoxville, Tennessee, United States of America; 2 Student Health Center, The University of Tennessee, Knoxville, Tennessee, United States of America; 3 Department of Environmental, Agricultural, and Occupational Health, College of Public Health, University of Nebraska Medical Center, Omaha, Nebraska, United States of America; 4 Department of Kinesiology, Recreation, and Sport Studies, The University of Tennessee, Knoxville, Tennessee, United States of America; 5 Department of Obstetrics and Gynecology, The University of Tennessee Graduate School of Medicine, Knoxville, Tennessee, United States of America; 6 United States of America Health Outcomes GlaxoSmithKline, Philadelphia, Pennsylvania, United States of America; 7 Department of Health, Behavior and Society, University of Texas School of Public Health San Antonio, San Antonio, Texas, United States of America; 8 College of Nursing, The University of Tennessee, Knoxville, Tennessee, United States of America; Regional Health Care and Social Agency of Lodi, ITALY

## Abstract

**Background:**

Over 47,000 human papillomavirus (HPV) related cancers are newly diagnosed every year in the United States, yet HPV vaccination rates are below desirable levels. Young adulthood is critical for catch-up HPV vaccination. Previous research on HPV vaccine-seeking behavior among young adults is limited, particularly in Tennessee. This study aimed to understand HPV vaccine perceptions and experiences of young adults.

**Methods:**

Virtual semi-structured interviews were conducted among 18–26-year-olds at a university in Tennessee from April to May 2022 (N = 25). Interview questions were based on the Health Belief Model and previous literature with a focus on gaining in-depth insights into young adults’ perspectives regarding HPV vaccination. Inductive and deductive thematic analysis approaches were used for data analysis.

**Results:**

Five major themes were identified: (1) perceptions about HPV and HPV vaccination, (2) factors that may prevent HPV vaccination, (3) role of health care providers, (4) role of family and friends, and (5) HPV vaccine promotion strategies. Participants commonly discussed lack of knowledge and awareness as a factor preventing HPV vaccine uptake. Participants described some facilitators of HPV vaccination, including healthcare provider recommendations and family support, and perceived future benefits of receiving the HPV vaccine. Suggestions for promotion strategies included promotion through college health centers, community education, and awareness campaigns.

**Conclusions:**

Young adults’ perceptions about the HPV vaccine and inadequate knowledge may influence uptake. Findings from this study showed that healthcare provider recommendations and parental support were facilitators for getting vaccinated. Future efforts may consider increasing awareness among young adults, exploring barriers to recommendation, and addressing parental concerns.

## Introduction

Human papillomavirus (HPV) causes almost 90% of cervical and anal cancers; 70% of oropharynx, vulvar, and vaginal cancers; and 60% of penile cancers [1]. Every year in the United States (U.S.), over 47,000 HPV-associated cancers are newly diagnosed [2]. These new diagnoses could be prevented, as evidence shows that the HPV vaccine is safe and effective [3]. The Advisory Committee on Immunization Practices recommends HPV vaccination from age 11 or 12 years but can be started as early as nine years [4]. HPV vaccination rates are below the Healthy People 2030 goal of 80% [5]. In 2022, only 62.6% of adolescents aged 13–17 years reported receiving the recommended dose of the HPV vaccine [6]. Catch-up HPV vaccination is routinely recommended for individuals who missed vaccination up to age 26 years [4]. However, only 22% of U.S. young adults aged 18 − 26 years reported completing the HPV vaccine series in 2018 [7].

Findings from previous research suggest several factors may influence HPV vaccine-seeking behavior among young adults. These factors include lack of knowledge and awareness, healthcare provider recommendations, perceptions related to susceptibility to HPV infection, and benefits of receiving the HPV vaccine [8–11]. Additionally, misconceptions about the HPV vaccine could also influence vaccination. For example, in one study among college students, the perception that getting the HPV vaccine may be considered too late was reported among participants [12]. Other reported factors influencing HPV vaccination among young adults include social stigma, parental or peer influence, concerns about the HPV vaccine, cost of the HPV vaccine, time constraints with college responsibilities, and lack of information regarding locations to obtain the HPV vaccine [9,11,12].

HPV vaccination coverage varies across regions in the United States. Findings from previous research have shown lower HPV vaccination coverage in Southern U.S. compared with other regions of the country [13–15]. Specifically for Tennessee, in 2022, about 74.1% of adolescents aged 13–17 years have initiated the HPV vaccine series, while about 64.4% completed the series [16]. These HPV vaccination rates are below the desired target set by Healthy People 2030 [5]. However, limited studies have focused on HPV vaccination behavior and experiences among adult population in Tennessee [11,17]. Additionally, few studies have utilized a qualitative approach to describe and capture young adults’ perceptions and experiences regarding HPV vaccination [8,12,18]. Understanding young adults’ perceptions and experiences about HPV vaccination is an essential step to improving vaccine uptake. Catch-up HPV vaccination for individuals up to age 26 is vital to achieving timely population-level impact of HPV vaccination [19]. Therefore, the objective of this study was to examine young adults’ perspectives regarding the HPV vaccination in Tennessee using qualitative approach. The research question that guided this study include: How would young adults describe their perceptions and experiences about the HPV vaccine? Findings from this study will provide insight into the motivation for HPV vaccine uptake. This information is necessary to inform future strategies to address barriers preventing HPV vaccine uptake.

## Methods

### Study design and subjects

This qualitative study utilized semi-structured interviews to gain in-depth insights into contextual factors associated with HPV vaccine uptake among students on a university campus in Tennessee. Eligibility requirements include undergraduate and graduate students between 18 and 26 years who were currently enrolled at the time of study (Spring 2022). Recruitment was done from March 31^st^ 2022 to May 6^th^ 2022. Potential study participants were recruited through email and flyer distribution through campus organizations and by posting flyers around popular campus buildings. For email recruitment, a list of email addresses of 300 currently enrolled students between 18–26 years was obtained from the university for research purposes. An email with study information and the link to sign up for the study was sent to the obtained email addresses. A second follow-up email was sent to those who did not respond to the first email invitation. A total of 60 individuals signed up for the study. Among those who signed up, eligible participants were contacted via email to schedule a time for an interview. All interviews were conducted from April to May 2022. The data collection process continued until no new information was obtained from the interviews. (i.e., data saturation) [20].

### Interview protocol

The interview guide was developed and adapted from previous research on HPV vaccination [21–24]. Interview questions were drafted to reflect the constructs of the Health Belief Model (HBM) [25]. The principle of HBM suggests that individual beliefs and perceptions likely influence the adoption of a health behavior [25]. The HBM comprises six key constructs that play a role in shaping an individual’s health-related behavior. These components include perceived severity, perceived susceptibility, perceived barriers, perceived benefits, cues to action, and self-efficacy [25]. Perceived severity describes a person’s beliefs about the gravity or impact of getting a disease [25]. Perceived susceptibility refers to individual beliefs about the chances of contracting a disease [25]. Perceived barriers refer to individual beliefs about hindrances to adopting a health behavior, including perceptions related to unfavorable effect of adopting the behavior [25]. Perceived benefits describe individual beliefs about the advantages of adopting a particular health behavior [25]. Cues to action refer to internal or external factors that stimulate the acceptance or adoption of behavior [25]. Lastly, Self-efficacy denotes a person’s belief in their capacity to perform a health behavior [25]. The HBM framework also acknowledges the role of modifying factors (demographic, structural, and psychosocial) related to health behavior change including age, race or ethnicity, sex, personality, socioeconomic status, and knowledge [25]. The interview questions focused on eliciting individual perceptions and beliefs that may influence HPV vaccine uptake. For example, results from a study among young adults by Jin and colleagues suggests that perceived barriers and perceived risk was associated with HPV vaccination among females [11]. Similarly, in a different study by Gerend et. al, perceived barriers and perceived susceptibility was associated with HPV vaccine uptake [26]. The interview questions are included as a supplemental file with this article.

The interview questions were reviewed for content and contextual appropriateness by experts in vaccination and college health research. Probes and follow-up questions were used to obtain detailed responses from participants. The interviews were conducted via Zoom in a private setting, with each session lasting about 16–53 minutes (mean = 30 minutes). Participants were asked to complete a brief (3–5 minutes) pre-interview questionnaire via Qualtrics (Provo, UT) to obtain sociodemographic and HPV vaccination information. Informed consent was obtained from participants online before administering the questionnaire and conducting the interview. One research team member conducted the interviews (O.O.), while another team member was present during the interviews to observe and provide feedback (C.N.). Detailed notes were taken during the interview and data collection procedures. Audio recordings and transcripts were automatically generated through Zoom Otter.ai transcription software (Mountain View, CA). Transcripts were reviewed manually for accuracy by a research team member (O.O.). Any personal identifying information was removed, and participants’ names generated in the transcripts were replaced with unique identification numbers. All participants who completed the interview received a $25 gift card.

### Ethical considerations

This study was approved by the University of Tennessee Knoxville Institutional Review Board (UTK IRB-22–07245-XM). Study participants completed a written informed consent before data collection. An IRB approved informed consent statement was attached to the beginning of the pre-interview questionnaire via Qualtrics. Participants were given adequate time to read the consent document and were encouraged to ask any questions regarding any aspect of the study. Participants who agreed to participate in the study were asked to select “I Agree” at the end of the informed consent statement.

### Data analysis

The data analysis for this study involved thematic analysis using both inductive and deductive approaches [20]. This involved the identification of themes that emerged during the interview (inductive), as well as using pre-determined themes based on HBM constructs and interview questions (deductive) [20]. Prior to data analysis, the research team met to discuss coding procedures. An initial codebook was created with pre-determined themes, and coders added additional themes as needed during the data analysis. Coding was an iterative process that involved the generation of common ideas shared by participants, as well as comparing and finding connections between themes by two of the researchers (O.O., C.N.). To ensure consistency in the analysis and interpretation of codes, the researchers met after coding the first three transcripts to discuss the coding process and any changes to the codebook. Thereafter, the two researchers (O.O. and C.N.) independently coded the transcripts and then met regularly to discuss themes and resolve conflicts. Decisions about unresolved disagreements were settled by a third research team member (K.W.K. or C.S.B.). Detailed notes about questions, findings, meanings, and thoughts that arose throughout the research process were recorded. The consolidated criteria for reporting qualitative research was referenced for this study [27]. NVivo software version 12 was used for data analysis, including computing interrater reliability which was 0.84.

## Results

### Participant characteristics

A total of 25 individuals participated in this study. The age of participants ranged from 19 to 26 years, with a mean of 21.2 years. Most participants identified as female (64%), White (72%), and non-Hispanic (64%). Most participants identified as heterosexual or straight (48%), were dating (60%), and resided in suburban areas (72%). All participants reported that they had heard about HPV, while 92% had heard about the HPV vaccine. About 68% reported that they had received the HPV vaccine. Table 1 presents the characteristics of study participants.

**Table 1:** Characteristics of study participants.

	Total		Vaccinated		Unvaccinated ^d^	
	N	%	n	%	n	%
**Sex**						
Female	16	64	11	64.7	5	62.5
Male	9	36	6	35.3	3	37.5
**Race**						
White	18	72	13	76.5	5	62.5
Others ^a^	7	28	4	23.5	3	37.5
**Ethnicity**						
Hispanic	9	36	6	35.3	3	37.5
Non-Hispanic	16	64	11	64.7	5	62.5
**Relationship Status**						
Single	9	36	6	35.3	3	37.5
Partnered ^b^	16	64	11	64.7	5	62.5
**Sexual Orientation**						
Heterosexual/Straight	12	48	6	35.3	6	75.0
Others ^c^	13	52	11	64.7	2	25.0
**Residence**						
Rural	4	16	2	11.8	2	25.0
Suburban	18	72	13	76.5	5	62.5
Urban	3	12	2	11.8	1	12.5

^a^Dating/Living with a romantic partner

^b^Black/African American/Asian/More than one race

^c^Gay/lesbian/bisexual/pansexual/queer

^d^Participants who reported ‘no,’ ‘unsure,’ or ‘don’t know’ to question about their HPV vaccination status

### Thematic analysis results

Data analysis revealed five major themes: (1) perceptions about HPV and HPV vaccination, (2) factors that may prevent HPV vaccination, (3) role of health care providers, (4) role of family and friends, and (5) HPV vaccine promotion strategies. Table 2 presents sample quotes for themes and subthemes.

**Table 2:** Themes/Subthemes and Sample Quotes.

Theme 1: Perceptions about HPV and HPV vaccination	
Perceived Susceptibility	“I am not currently sexually active, so I would not consider myself at risk…Well, I thought of it, one day, I would like to be sexually active if I get a boyfriend etcetera….So I would like to be protected if I were to contract it from somebody, a new partner that’s kind of my thought process of it, even though I’m not at risk, now, I would like to be protected in the future” *(p107, female, vaccinated, three doses)* “...because I’ve heard about HPV but I’ve never known like anybody that’s ever gotten it or any infections related to it, and so I just kind of feel like I’m kind of in the same like it, like it won’t happen to me either” *(p110, male, unvaccinated)*
Perceived Severity	“I think it would be pretty devastating like it sounds super scary and it sounds like it just to be life changing, and so I definitely think the issues, probably pretty serious but since I just don’t see it a lot I don’t really end up thinking about it, too often, but I do think that it would cause some serious lifestyle and health risks” (*P109, female, unsure about vaccination status)*
Perceived Benefit: *Cancer Prevention*	“ I do believe that I am protected against the various strains that would make one susceptible of developing any cancers in the future Even though I don’t consider myself to be in or having a lifestyle that would put me at risk, but I do think it is very beneficial for preventing cancer and developing in the later stages of life” *(p156, male, vaccinated, two doses)*
Perceived Benefit: *Peace of Mind*	“I would say the major benefit other than prevention of HPV is peace of mind and i do feel like it’s one of those things where it’s like okay, I have been vaccinated for this, I can rest a little bit easier and not have to worry” *(p104, female, vaccinated, three doses)*
Perceived Benefit: *Future Protection*	“…if you’re in my case and just single You would still want to get it because yeah sure you’re not in a relationship now but in the future, there is always that possibility of either yourself getting HPV from somebody else, or you giving it to others” *(p116, male, vaccinated, two doses)*
Perception related to priority or sense of urgency	“Personally, I don’t believe I’ve had any barriers, the main thing is just my it’s my it’s my effort to actually go out and do it…I guess just I just it was not a priority for me, I think” *(p134, female, unvaccinated)* “I just didn’t see any need to get it, as I mentioned, I wasn’t active didn’t see a need to get it at that moment [*previously not vaccinated*], so it wasn’t anything. Like I wasn’t scared of it or anything I just didn’t feel the need to get it to…” *(p107, female, vaccinated, three doses)*
**Theme 2: Factors that may prevent HPV vaccination**	
Lack of knowledge and awareness	“…I feel like the biggest barrier would be just me not knowing that there was one that existed. You know, so I guess lack of knowledge, probably is the biggest one” *(p127, female, unsure about vaccination status)* “Honestly, I haven’t heard much about it except for that it’s good to be vaccinated it wasn’t necessarily something that I was told a lot about before I got the vaccine…” *(p125, female, vaccinated, two doses)*
Lack of time	“I would say the biggest one is just making time to go receive it, because it was a completely separate appointment that I just had to go back to get that and yeah so it was just finding time when it felt kind of like a chore and I didn’t really know how urgent it was like it felt like just like Oh, if I miss it I miss it like whatever so yeah That was the biggest thing just time” *(p111, female, vaccinated, unsure about number of doses)*
Cost and lack of insurance	“Maybe the price of it if you can’t afford it, or if you’re tight on money that might not be something that you spend your money on” *(p161, female, unsure about vaccination status)* “…the only times I’ve ever like been somewhere to like get the shot or heard anything about it really has been in a doctor’s office, and if you can’t afford to go the doctor, like if my parents would not have been able to afford to take me to the doctor then I would have never heard about HPV vaccine and ever had an opportunity to get it. So, I guess like If you aren’t like going to the doctor or anything like you probably haven’t heard of it. Maybe if you’re not able to afford a phone you maybe won’t see it pop up like on the news or anything.” (p110, male, unvaccinated)
Stigma	“So when I was younger my mother didn’t want me to have it Mostly, because I think we come from a fairly conservative background and so her thought was, you know that that the risk of getting HPV is due to perceived promiscuity, or having multiple partners and so she thought you know if you’re only ever going to have one partner and they’re only ever going to have one partner, then there’s no risk of it And since she felt like the vaccine was newer as well, and she didn’t know all of the risks she opted for me not to get it and I actually just last year had a conversation with a physician now because I’m 26 and able to kind of make more decisions for myself so I’ve just started…” *(P130, female, vaccinated, unsure about number of doses)* “...I don’t know much about the stigma, but in my mind I would think that that would be a thing, especially for mothers and their daughters just thinking about them being sexually active.” *(p134, female, unvaccinated)*
Concerns about side effects	“...In terms of the perception of side effects, that was a big barrier for my mom for not getting it for me when I was younger I was just worried that something could come up down the line, or we find out that secretly everybody that got this vaccine is you know infertile or develops some other thing so I think there’s definitely hesitancy around it for those reasons, even if they’re not necessarily named side effects just the idea that there could be side effects was enough for her to worry” *(P130, female, vaccinated, unsure about number of doses)*
Fear of needles	“I haven’t really had many barriers that have prevented me from the vaccine like I’ve had multiple opportunities to get it, I would say, I guess, the only barrier is just like. I just don’t like unnecessary shots just because I don’t like needles, so I guess the only barrier would be that I just dislike shots if it wasn’t for that I probably would have gotten it by now.” (p110, male, unvaccinated)
Misinformation	“So, at first, they were all for it and I got the first one, and then I think it was my step mom or my dad one of them found some article about how it was bad for you, and it was causing all these issues for women and things like that so, then they were like we’re not going to get you the second one, so at first, they were okay with it, and then they were against it for a while and they still are against it” *(p119, female, vaccinated, two doses)* I mean there’s always like. anti vaxers who are like against vaccines, like, I guess, like fake news and like fake posts and untrue articles with negative side effects in it, or like put it in a negative stuff could probably keep people away from it. *(p110, male, unvaccinated)*
Healthcare system accessibility and administrative barriers	“…there’s a lot of factors, but I think the main one is that lot of people in the United States don’t see a doctor regularly. This is for a variety of reasons, it could be like kind of you know, personal distrust of the medical community or it could be, because their family just doesn’t or they don’t have good insurance, and so they don’t really know they get stuck in the bureaucracy of our inadequate medical system and end up just kind of neglecting their own personal health…” *(p106, male, vaccinated, two doses)* “While my physician was on board, she was kind of she had some difficulty pulling some of my old records and I had to like get in contact with all of my old providers and try to get less than over to her of all my vaccinations which was You know, it might not have been a barrier to me, necessarily, but I could see that being a barrier to someone else who didn’t have like as long of a Health history” *(P130, female, vaccinated, unsure about number of doses)*
Lack of adequate follow-up	“I got one dose when I was very young, I want to say I am now 22 I got vaccinated when I was 14 and I need to go back and I probably needs to finish it off or restart the process, because I only got one shot and three shot process and it was fine but I’m a military brat we moved immediately afterwards and then kind of forgot about it” *(p102, female,vaccinated, one dose)*
**Theme 3: Role of Health Care Providers**	
Role of Health Care Providers	“…it was just something that my health care provider brought up to me and my guardian at the time we were both in the room I think it was just a regular checkup appointment and they just recommended that I get the vaccine for general health reasons, and I trusted my health care provider and my guardian and also recommended that I get it…” *(p125, female, vaccinated, two doses)* “...If he like told me that like it’s like life or death, I had to get it, I would probably get it or if he told me that it would like significantly helped me like health wise, I would get it. Just because, like he’s a doctor and he knows more than I do so I’d probably do it” *(p110, male, unvaccinated)* “…if my doctor had mentioned that I had not gotten the full effect of the HPV vaccine. I would have gone back and done it probably that day at the office, if it was available” *(p102, male, vaccinated, one dose)*
**Theme 4: Role of Family and Friends**	
Role of Family and Friends	“My parents encouraged me to take it before I went to college, I believe, and I was you know agreed. so I yeah I ended up taking it...” *(p114, female, vaccinated, one dose)* “… I think my mom just advised me to deny it, because it’s like assumed that I won’t be like engaging in like any sexual activity or anything so I’ve heard that people or parents say no, on behalf of their child because they don’t know or they believe their child won’t like engage in any like sexual activity, but I think it’s just preventative” *(p147, female, vaccinated, two doses)* “I would probably say if my friends and family pressured me into getting a vaccine or told me about like how the vaccine has been beneficial for them It might lead me to want to get it.” *(p110, male, unvaccinated)*
**Theme 5: HPV Vaccine Promotion Strategies**	
Increase accessibility/promoting accessible vaccination options	“I think that if it was widely offered on campus which I haven’t looked into, if it is or not, but if it was available at the student health center and promoted maybe on the university student life page or something like that, and like an information session about it, offering it either for like reduced cost of insurance or like for free I think that would encourage a lot of people to be able to go get it, because if they live around campus or they’re here for classes, they can use walk there easily and it doesn’t take that much time out of their day to do so” *(p111, female, vaccinated, unsure about number of doses)* “I think probably like student health center, like being like I don’t know like saying like you can get it done here at this time, but make it like easier to like physically get it …” (*P109, female, unsure about vaccination status)* “if it was more accessible, maybe if we could get it like a less formal environment like how it’s a lot easier to get like a flu shot or covid vaccine than it is to get like other vaccinations that like you’re supposed to get like certain age milestones I guess. So, I would say, just like an increased access to it, like you can just go through a drive through like you could with covid vaccines. It will probably help people if they know they wouldn’t have to like sit at a doctor’s office all day to get it” *(p110, male, unvaccinated)*
Community awareness campaign and education	“On campus there could be multiple ways that you could just promote getting the vaccine. It could be flyers everywhere, I look are on every board somewhere there’s a flyer or something for an event happening or it’s some type of PSA or something along those lines or even holding an event in itself and then promoting the said event for people to get the vaccine like HPV awareness day…I feel like getting that information out there to more and more people on campus would definitely influence their decision and getting a vaccine” *(p116, male, vaccinated, two doses)* “I would say, by putting stuff to raise awareness like on billboards like on the side of the road, maybe, or just maybe like putting flyers like in homeless shelters and I guess pushing harder to spread awareness about it.” *(p110, male, unvaccinated)*
Information preferences	“I think hearing about it and in general would be helpful for anybody who doesn’t necessarily know about it and hearing the data, but in a in a very easily understood and palatable way, because sometimes with a lot of things, a lot of data will just be shown and it’s kind of overwhelming and so you just decide to come back to it when you have the time to deal with making that decision, but you know, providing all the information needed in a easily understood way makes people feel less overwhelmed” *(p125, female, vaccinated, two doses)* “I think if It’s [*HPV vaccine] …* being offered verbally by my health care provider or just word of mouth aspect makes a big difference, I think in my opinion, and just hearing the importance of it [*HPV vaccine]* would really convinced me to.” *(p134, female, unvaccinated)*

### Theme 1: Perceptions about HPV and HPV vaccination

#### Perceived susceptibility.

Perceived susceptibility captures participants’ opinions regarding their risk of getting infected with HPV or being diagnosed with HPV-related cancers. Findings related to perceived risk were not unanimous among participants. Some participants discussed during the interviews that they did not consider themselves at risk because they were not currently sexually active or not in a relationship. Interestingly, other participants highlighted the need to be protected against HPV in the event of future relationships that may put them at risk. Another reason that some participants did not consider themselves at risk was being in monogamous or committed relationships, use of protection, and regular HPV testing/annual checks. A few participants felt that the presence or absence of a family history of cancer made them more or less at risk of cancer, respectively. Some participants also shared that a lack of knowledge about HPV prevented them from determining whether they were at risk of HPV infection.

#### Perceived severity.

This subtheme captures discussion related to participants’ perceptions about the seriousness and negative impact of HPV infection and HPV-related cancer. Most participants agreed that being diagnosed with HPV-related cancer would have a severe negative impact on their lives. The majority of participants were not particularly concerned or worried about the negative impact of HPV infection because they had received the HPV vaccine. However, some participants felt they did not know enough about HPV to determine the extent to which they would be affected by HPV-related cancers.

#### Perceived benefits.

This subtheme describes participants’ beliefs and opinions regarding the benefits of receiving the HPV vaccine. The ideas and perspectives related to benefits included cancer prevention, peace of mind, and future protection. Most participants believed the HPV vaccine would protect against cancer. However, some participants expressed lack of knowledge about the benefits of the HPV vaccine. A commonly reported benefit of the vaccine was peace of mind. Participants explained that getting vaccinated for HPV made them feel they would not have to worry about the possibility of getting HPV infection or HPV-related disease. Several participants talked about how receiving the HPV vaccine offers future protection against HPV. While some participants did not feel that they were currently at risk of HPV infections, they were motivated to receive the vaccine because they believed lifestyle changes or future relationships may predispose them to contracting HPV. Another benefit of receiving the HPV vaccine was protecting their future partners in addition to protecting themselves. A few participants expressed lack of perceived sense of urgency about the vaccine and therefore, it was not a priority for them.

### Theme 2: Factors that may prevent HPV vaccination

This theme describes participants’ perceptions and experiences regarding factors that prevent HPV vaccination. Most participants who had been vaccinated reported minimal barriers to receiving the HPV vaccine. This was largely attributed to several facilitators of HPV vaccination such as parental support/decision, having health insurance, attending annual clinic visits, and healthcare provider recommendations. Participants who reported having no specific barriers to vaccination were asked to discuss barriers among young adults like them.

Lack of knowledge and awareness was commonly reported, with several participants expressing that they did not know much about the HPV vaccine. In terms of actual barriers preventing vaccine uptake, one participant expressed dislike of needles as a barrier. Lack of regular access to healthcare was highlighted as a factor preventing completion of the HPV vaccine series. Others expressed logistical barriers, such as finding time to drive to the doctor’s office in the presence of competing school activities. Cost was also discussed as a barrier, particularly in the context of lack of insurance. Both lack of time and cost were barriers to receiving multiple doses and requiring more than one appointment to receive the HPV vaccine, which may influence the uptake of the second or third doses of the HPV vaccine. Another barrier shared by a participant was a negative experience when transferring medical records from pediatric to adult care that made it difficult to access HPV vaccination status/records. Several participants discussed stigma related to HPV infection as a sexually transmitted illness (e.g., parental perceptions of promiscuity associated with HPV vaccination) and misinformation or concerns about vaccine side effects.

### Theme 3: role of healthcare providers

This theme details participants’ discussion related to how their healthcare provider stimulated HPV vaccine uptake. Most participants acknowledged the influential role of their healthcare provider recommendations in their HPV vaccine decision-making process. Some participants mentioned that a combination of their parent/caregiver and doctor’s influence motivated them to receive the HPV vaccine. Several participants noted that their healthcare provider is their preferred source of HPV vaccine information. Participants explained that they trust the recommendations regarding the HPV vaccine when provided by their healthcare provider. For one participant, having a consistent patient-provider relationship was important to build trust. Similarly, another participant discussed that incorporating HPV vaccine recommendations into routine doctor’s visits was important to their HPV vaccine decision-making. Other perspectives shared by participants regarding the role of providers included perceived lack of a sense of urgency and importance during discussions about HPV vaccination and the need for reminder emails to prompt individuals.

### Theme 4: role of friends and family

This theme captures common ideas related to perceptions, attitudes, and influence of family and friends towards HPV vaccination. Findings related to family and friends’ influence on HPV vaccine decision-making were mixed. While some felt that their family or friends’ attitude (negative or positive) towards HPV vaccination was influential, others expressed that they make personal health decisions using their own research or that they would ultimately trust their doctor’s advice over family or friends. Notably, one participant decided to receive the HPV vaccine at the age of 18 years even though parental refusal had prevented uptake of the vaccine as an adolescent. A commonly expressed thought among participants who have been vaccinated as an adolescent or before college was that parental/caregiver support or decision made them get the HPV vaccine.

### Theme 5: vaccine promotion strategies

#### Increase accessibility/promoting accessible vaccination options.

A common suggestion among participants was to promote the HPV vaccine through the university student health services. Some respondents suggested HPV vaccine information can be promoted by the student health services to those presenting for care, including the use of posters around the clinic areas. Participants felt that a key benefit of promoting availability through student health services was the ease of getting the vaccine due to the proximity of the health center to campus. Students would not need to find time to drive longer distances to their doctor’s office. However, concerns related to cost and affordability were raised, especially for uninsured individuals. Participants suggested that student health services could partner with student organizations such as sororities and fraternities to promote HPV vaccination. Participants highlighted alternative ways to increase accessibility including vaccination at pharmacies, free clinics, and drive-through options. Reducing disparities in access to healthcare was also discussed as this may encourage regular doctor visits and, ultimately, routine preventive care such as vaccination.

#### Community awareness campaign and education.

Several participants expressed the need for HPV vaccine education and creating more awareness. Examples discussed by participants include tabling events or vaccination booths at popular campus locations, incentives, social media campaigns, promotion emails through university listservs, and use of flyers and posters around campus buildings. A few participants discussed the promotion of the HPV vaccine to adolescents, for example, incorporating HPV vaccine education in high school settings.

#### Information preferences.

Participants expressed their preferred type of educational information related to HPV infection and HPV vaccination. These included statistics related to the prevalence of HPV infection and vaccine efficacy, benefits of HPV vaccine, risk factors for HPV, vaccine side effects, vaccine schedule, and vaccine locations. Participants also highlighted sources of HPV vaccine information. Commonly shared sources of HPV vaccine information by participants included internet searches, healthcare providers, pharmacies, social media, and health departments. Some participants expressed the need for more information from trusted sources such as healthcare or public health institutions, especially in a simple and easy to understand format.

## Discussion

This study explored young adults’ perceptions and experiences regarding HPV vaccination. Findings from the study revealed five major themes, including perceptions about HPV and HPV vaccination, factors that may prevent HPV vaccination, role of healthcare providers, role of family and friends, and HPV vaccine promotion strategies.

A noteworthy observation from our results was the discussion related to inadequate knowledge or awareness about the HPV vaccine. This finding is consistent with other qualitative studies. In a study by Meta and colleagues, focus groups were conducted among men to explore HPV vaccine acceptability [8]. The study found a lack of knowledge as a common theme, which may also translate to other beliefs or perceptions. In other words, participants may not have adequate knowledge to evaluate susceptibility to HPV and weigh the benefits of getting vaccinated against the severity of HPV infection and HPV-related cancer or overcoming barriers to getting vaccinated. This lack of knowledge shows the need for increasing education and awareness among unvaccinated young adults. Future HPV vaccination efforts should incorporate young adults’ information preferences to increase education and awareness. Examples may involve promoting credible internet sources and social media campaigns.

Another notable finding from this study was the influential role of healthcare provider recommendation in HPV vaccine decision-making. Participants commonly discussed that having personal conversations with their healthcare provider was a strong motivation for receiving the HPV vaccine. For some, these conversations often involved discussion between their parents/caregivers and doctors as adolescents. The influential role of healthcare providers may be due to several participants’ belief that their healthcare provider was a trusted source of HPV vaccine information. Previous studies have suggested that individual or parental trust in their healthcare provider was associated with HPV vaccine uptake or intention to receive the vaccine [28–30]. Hence, it is important for healthcare providers to foster or leverage established patient-provider relationships to increase HPV vaccine recommendation and uptake.

While previous studies have demonstrated a strong association between provider recommendation and HPV vaccine initiation and completion [31,32], recommendations may not be consistently provided across different patient populations [33]. Barriers to healthcare providers recommending the HPV vaccine may occur at different levels, including individual level (e.g., lack of provider knowledge or confidence, low quality and strength of provider recommendation), clinic level (e.g., time constraints, lack of reminder and recall system), and policy level (e.g., practice guidelines, school requirement) [34]. Future HPV vaccination strategies may consider exploring opportunities to address hindrances preventing recommendations by healthcare providers.

Another influential factor for vaccination described among participants was parental or caregiver approval and support. This corroborates findings from previous research that showed an association between the perception of approval from family and friends and HPV vaccination among adults on a college campus [35]. This depicts the importance of addressing barriers preventing family acceptance or approval of HPV vaccination. For example, a previous study based on data from the 2010–2016 National Immunization Survey – Teen reported several parental reasons for refusing to vaccinate their female children, which included safety concerns, beliefs that vaccination is unnecessary, perceptions that the child is sexually inactive, lack of knowledge and not receiving provider recommendations [36]. Safety concerns as a reason for not vaccinating persisted among female parents and increased among male parents from 2010 to 2016 [36]. With substantial research demonstrating the safety, efficacy, and long-term benefits of HPV vaccination [3], innovative communication approaches may be needed to debunk misconceptions related to safety concerns. Furthermore, it is important to consider targeted catch-up HPV vaccination campaigns and programs for young adults who may have missed HPV vaccination in childhood due to parental refusal.

One approach for increasing HPV vaccination among adults on college campuses identified by participants was promotion through college or university student health services. University or college campuses present a unique setting for health promotion among young adults including improving HPV vaccination [37]. Healthcare providers that serve college student populations at university student health services are uniquely positioned to provide education and effective recommendations regarding the HPV vaccine since they already offer related healthcare services [38,39]. The College Health Surveillance Network conducted a study on healthcare utilization by college students across 23 four-year universities in the United States over a 41-month period [40]. The study found that there were 4.17 million clinical visits made by 802,255 individuals to the university student health center [40]. Of these visits, 60% were for primary care, 13% for mental health, 9% for vaccinations, and 13% for other reasons [40]. College students may also view their school’s health center as their source of routine care. For example, in a previous study among students in a southeastern university, approximately 56% reported receiving care from a healthcare provider at their institution’s student health center within the past year [41]. In the same study, 47% of participants reported that they would seek both preventive and acute care for illness at their university’s student health center [41]. Despite these findings, students may not be familiar with the opportunity to vaccinate at student health centers. About 48% of college students in one study reported being unaware that the HPV vaccine was provided by their school’s health center [42]. This suggests the importance of improving awareness about the availability and accessibility of HPV vaccination on college campuses with health centers that administer the HPV vaccine. Additionally, healthcare providers serving college students may consider leveraging opportune clinic visits to assess and recommend HPV vaccination.

A few of the participants in this study were unsure about their HPV vaccination status. This may be related to the transition of vaccination records from pediatric to adult care. Findings from the interviews reflect logistical challenges associated with transferring pediatric records to primary care providers to facilitate vaccination. This is particularly important for individuals who are within the HPV vaccination catch-up age group who are not aware of their vaccination status. Immunization information systems can be important facilitators to ensure vaccination records are available for patients and providers as children transition into adulthood [43]. However, several barriers have limited the widespread use of such systems across the U.S. [44].

## Strengths and limitations

This qualitative study provide useful insights into the perceptions and experiences of young adults regarding HPV vaccination on a college campus in Tennessee. Study findings will be useful for the development and implementation of HPV vaccination intervention among young adults. This study has a few limitations. First, all study participants have heard about HPV, and most have heard about the HPV vaccine. Hence, our participants’ perspectives may differ from those who have not heard about the HPV vaccine. Second, this study was conducted during the COVID-19 pandemic. Hence, participants’ perspectives and experiences about HPV vaccination may vary before and after the pandemic. Third, our study population was mostly vaccinated which may reflect discussions related to positive perceptions about the HPV vaccine. Despite these limitations, trustworthiness of the data was ensured in different ways, including providing a thick description of study procedures, keeping detailed notes on study procedures and findings, regular team meetings, and peer debriefing [45].

## Conclusions

Healthcare provider recommendation was discussed as a facilitator for HPV vaccine uptake in this study. Future studies may consider examining barriers preventing timely and effective HPV vaccination recommendations. Future research may also consider exploring how sociodemographic characteristics such as sex, race/ethnicity, sexual orientation, and geographical residence influence HPV vaccination decision-making. Additionally, future HPV vaccine education programs should consider incorporating evidence for vaccine safety and effectiveness over the years. Lastly, given the limited availability of evidence-based interventions to improve HPV vaccination rates among college or university students, future HPV vaccine intervention should partner with college or university health services, since they are uniquely positioned to reach HPV vaccination catch-up population.

## Supporting information

S1 Appendix Interview Questions. (DOCX)
