# Supplementary material for: Understanding young adults’ perceptions regarding human papillomavirus vaccination: A qualitative study
Source: PLoS One. 2025 May 12;20(5):e0323063. doi: 10.1371/journal.pone.0323063 (PMC12068637; doi:10.1371/journal.pone.0323063)
Supplement: S1 Appendix — (DOCX) [file pone.0323063.s001.docx]

**Interview Guide**

1. What have you heard about HPV and the HPV vaccine?
   1. Tell me about your experience with the HPV vaccine?
2. Do you consider yourself at risk for HPV infection or HPV-related cancer? Why or why not?
3. How do you think contracting an HPV infection or HPV-related cancer will affect you?
   1. Do you worry about HPV infection or HPV-related cancer? Why or why not?
4. In your opinion, do you feel the HPV vaccine will prevent you from getting cancer?
   1. What benefits do you think you will receive for getting the HPV vaccine? (If unvaccinated)
   2. What motivated you to receive the HPV vaccine? (If vaccinated)
5. Could you tell me some of the barriers that you feel have prevented you from being able to receive the HPV vaccine?
   1. Please describe the specific barriers, Transportation? Costs? Side effects?
   2. What are some of the barriers that you encountered when you decided to get the HPV vaccine? (If vaccinated)
6. How would you like to receive recommendations about the HPV vaccine? (Social Media? TV? Healthcare provider? Pharmacy? Public Health Organizations? friends/family?)
   1. What information would you consider useful?
7. Tell me what it would take for you to get the vaccine? (If unvaccinated) OR Tell me how you decided to take the vaccine (if vaccinated)?
   1. Do you feel you have/had everything that you need(ed)?
8. How do you think your social network or community (e.g., social groups on campus, family) feel about the HPV vaccine?
   1. How does their opinion impact your decision to receive or decline the vaccine?
9. What do you think will encourage individuals like you to receive the HPV vaccine? What would make the process easier?

*Conclusion*

Outside of what we have discussed, is there anything else you would like to share? Thank you for volunteering to participate in this study.
